# The Effects of Arginine, Guanidinoacetic Acid and Citrulline Supplementation to Reduced Protein Diets for Aged Laying Hens

**DOI:** 10.3390/ani16111664

**Published:** 2026-05-29

**Authors:** Aamir Nawab, Thi Hiep Dao, Sukirno Sukirno, Nasima Akter, Eunjoo Kim, Tamsyn M. Crowley, Amy F. Moss

**Affiliations:** 1 School of Environmental and Rural Science, Faculty of Science, Agriculture, Business and Law, University of New England, Armidale, NSW 2351, Australia; 2 Faculty of Animal Science, Vietnam National University of Agriculture, Gia Lam District, Trau Quy Town, Hanoi 100000, Vietnam; 3 Faculty of Animal Science, University of Mataram, Jl. Majapahit 62, Mataram 83125, Lombok, Nusa Tenggara Barat, Indonesia; 4 School of Life and Environmental Sciences, Faculty of Science, The University of Sydney, Camperdown, NSW 2006, Australia; 5 Poultry Research Foundation, The University of Sydney, Camden, NSW 2570, Australia; 6 Poultry Hub Australia, University of New England, Armidale, NSW 2351, Australia

**Keywords:** reduced protein diet, laying hens, enzymes, nutrient digestibility

## Abstract

This study evaluated the effects of arginine (Arg), guanidinoacetic acid (GAA), and citrulline (Cit) supplementation in reduced protein (RP) diets on the production performance, serum uric acid level and bone quality of aged laying hens. A total of 208 Hy-Line Brown laying hens from 60 to 75 weeks of age were assigned to eight dietary treatments, with 13 replicate cages per treatment and two hens per cage. Experimental diets comprised two protein levels (standard protein at 15.8% crude protein versus RP at 13.8% crude protein), and six RP diets supplemented with two inclusion levels (0.06% and 0.12%) of Arg, GAA, or Cit. Overall, increasing Cit supplementation improved productive performance compared with Arg and GAA, particularly by increasing egg weight (*p* = 0.048), egg mass (*p* = 0.019), and feed intake (*p* = 0.014) over the entire experimental period. In addition, Cit increased egg weight during 60–67 weeks (*p* = 0.049) and improved egg mass during 68–75 weeks (*p* = 0.014). Furthermore, supplementation of 0.12% Cit in RP diets significantly increased feed intake compared with standard protein (SP) and RP diets from 60 to 67 weeks (*p* < 0.001; one-way ANOVA). Both Cit and Arg supplementation increased yolk weight compared to GAA (*p* = 0.018). Further, Arg supplementation to the RP diet at 0.06% significantly increased energy digestibility compared to the SP and RP diets (*p* = 0.037). Increasing Arg and GAA levels reduced bone breaking strength compared with their respective low levels, whereas increasing Cit level enhanced tibia (*p* = 0.014) and femur (*p* = 0.006) bone breaking strength. Low-level GAA supplementation also increased tibia breaking strength. Thus, a moderate reduction in dietary protein level by two percentage points, combined with Cit supplementation at the high level (0.12%), was effective in enhancing egg mass, egg weight, yolk weight, and bone breaking strength in aged laying hens. These findings suggest that RP feeding strategies supplemented with functional Arg precursors may support productive efficiency, skeletal health, and nutrient utilization in late-lay hens.

## Simple Summary

This study examined how adding arginine, guanidinoacetic acid, or citrulline to reduced protein diets affects performance and bone health in older laying hens. The results showed that citrulline supplementation, especially at a higher level (0.12%), improved feed intake, egg weight, and egg mass more consistently than arginine or guanidinoacetic acid. Citrulline and arginine increased yolk weight and only citrulline significantly strengthened bones compared to guanidinoacetic acid. Arginine improved energy digestibility, but it did not enhance egg production or bone strength as effectively as citrulline. Overall, reducing dietary protein slightly and supplementing citrulline was the most effective strategy for improving egg quality, and bone strength in aged laying hens.

## 1. Introduction

Protein remains a critical nutrient in laying hen diets because of its essential role in growth, feed efficiency, and egg production [1]. Reducing crude protein (CP) levels in poultry diets has gained increasing attention due to its potential environmental and economic benefits. Lower dietary CP can reduce nitrogen excretion and ammonia emissions from poultry production systems, thereby improving bird welfare and reducing environmental pollution [2,3]. Ammonia is a major environmental pollutant that negatively affects poultry health by impairing respiratory function, damaging epithelial tissues, and reducing productive performance, while also contributing to odor generation and surface water eutrophication [4,5,6]. In laying hens, reduced CP diets have also been associated with lower water consumption, improved litter quality, enhanced nutrient utilization, and reduced disease incidence [7]. However, the increasing cost and limited availability of conventional protein ingredients, particularly soybean meal, have become major constraints in poultry production, where feed costs may account for 60–70% of total production expenses [8,9,10]. These challenges have encouraged the development of more efficient nutritional strategies, including reduced protein (RP) diets supplemented with crystalline amino acids (AA) and the exploration of alternative feed resources such as Azolla, insects, and microalgae for livestock and poultry production [11,12]. The importance of developing sustainable and cost-effective feeding strategies continues to grow as eggs and poultry meat remain major sources of dietary protein globally.

In Australia, egg consumption increased from 263 eggs per capita in 2022–2023 to 266 eggs in 2023–2024, and this trend is expected to continue [13]. Similarly, the global egg industry generated approximately USD 248.49 billion in 2022 and USD 270.08 billion in 2023, reflecting continued industry expansion [14]. In response to this growing demand and the need for more sustainable production systems, maintaining AA balance in RP diets through supplementation with crystalline amino acids (AA), including arginine (Arg) or its metabolic precursors, has been shown to fulfill Arg requirements, improve nitrogen utilization, and support productive performance in laying hens [15,16]. However, the extent of CP reduction must be carefully managed, as excessive reductions may negatively affect growth and performance outcomes [17]. Emerging evidence indicates that overly low CP levels in diets can impair feed efficiency and productivity in laying hens [18,19]. For instance, Shim et al. [20] reported that hens fed diets with a 4% reduction in CP, even with essential AA supplementation, showed reduced productivity between 18 and 74 weeks of age. Similarly, Gunawardana et al. [21] observed decreased egg weight in hens fed a 15.5% CP diet compared with those fed 16.1% CP during the 76–87 week laying period. Therefore, determining optimal protein reduction thresholds and precisely balancing synthetic AA supplementation are essential to maintain laying performance, particularly in aged laying hens. The global laying hen industry may benefit from the adoption of reduced protein diets supplemented with synthetic AA in terms of economic efficiency, environmental sustainability, and animal welfare [22].

Crystalline Arg, guanidinoacetic acid (GAA), and citrulline (Cit) are commercially available sources of Arg used in poultry nutrition [23]. Chickens are unable to endogenously synthesize Arg due to an incomplete urea cycle and the absence of key enzymes required for Arg biosynthesis [24]. Consequently, chickens rely entirely on dietary sources to meet their Arg requirements. Crystalline Arg and its metabolites are considered to have relatively high digestibility and bioavailability [25,26], compared with protein-bound arginine. Arginine acts as a precursor to creatine, polyamines, nitric oxide (NO), ornithine, and Cit pathways involved in collagen synthesis, wound healing, gut development, immunity, and macrophage activation [27,28]. In laying ducks aged 17 to 31 weeks, feeding 0.66 to 1.46% Arg diet increased egg weight, shell percentage, shell thickness, and yolk color score at 1.46%, and enhanced yolk percentage at 1.26% [29]. Yuan et al. [30] also observed that laying hens (Xinyang black) fed increased levels of Arg (1.25%, 1.26%, or 1.27%) had improved feed intake, egg weight, laying rate and feed efficiency from 33 to 45 weeks of age.

In chickens, dietary Arg reduces lipid peroxidation and superoxide emission, while improving antioxidant status and overall performance [31,32]. In birds, GAA is endogenously synthesized in the liver and kidneys from glycine and Arg, serving as a precursor for creatine synthesis [33]. Arginine acts as a guanidino-group donor in the formation of GAA, making it a key substrate in creatine biosynthesis. Therefore, dietary supplementation with GAA can reduce the metabolic demand for Arg in creatine production, thereby sparing it for protein synthesis and other physiological functions. In addition to its functional role, GAA presents economic and practical advantages as it is less expensive and more chemically stable than both Arg and creatine, making it a promising feed additive. Guanidinoacetic acid has also been associated with improved energy homeostasis, growth rate, and feed efficiency in poultry [34]. Furthermore, previous studies have reported that dietary GAA supplementation enhanced tibia mineral density and breaking strength in broilers, further supporting its potential benefits in poultry diets [35]. Similarly, Cit, a metabolite of Arg, can spare Arg in poultry despite not being directly incorporated into protein synthesis. As an intermediate of the urea cycle, Cit can be converted back to Arg through argininosuccinate synthesis, thereby contributing to arginine regeneration and nitrogen homeostasis. Previous studies have shown that dietary Cit supplementation in laying hens could enhance Arg utilization, promote nitric oxide synthesis, boost antioxidant defenses, and increase egg shape index [36,37]. While the effects of various Arg sources, including crystalline Arg, GAA and Cit, have been well documented in broilers and broiler breeders, limited research has focused on their application in aged laying hens maintained on reduced protein diets. Aged laying hens exhibit altered nutrient metabolism, reduced nutrient utilization efficiency, and declined productive performance during the late laying phase, making nutritional optimization particularly important under RP feeding strategies. Therefore, this study was designed to assess the effects of dietary supplementation with Arg, GAA, and Cit in RP diets on laying hen performance, egg quality, nutrient digestibility, serum uric acid level, and bone quality during the late laying phase.

## 2. Materials and Methods

### 2.1. Birds and Animal Husbandry

This study was implemented at the Laureldale Research Station at the University of New England, Armidale, New South Wales, Australia. Animal ethics approval was granted by the University of New England Animal Ethics Committee (ARA23-074). The study was completed according to the Australian Code of Practice, and the requirements of bird care and use for scientific purposes were met [38]. In total, 208 Hy-Line Brown (*Gallus gallus domesticus*) laying hens were obtained from a local commercial layer farm in Tamworth, New South Wales, Australia. All birds were from the same flock and were reared in cages according to the Hy-Line Brown management guidelines [39]. Upon arrival at 58 weeks of age, the birds were randomly distributed into 104 cages (two birds per cage) in a curtain-sided shed, with cage dimensions of 45 cm height × 50 cm depth × 30 cm width. Upon arrival, birds were fed a common commercial diet for two weeks until the study started (Barastoc-Premium Top Layer Mash: 16.5% CP, 2.5% crude fat, 6% crude fibre, copper 8.0 mg/kg, selenium 0.3 mg/kg, 3.6% calcium, and 0.3% salt, Melbourne, Victoria, Australia). Hens had ad libitum access to feed and water throughout the study duration via the provision of one feed trough and two nipple drinkers per cage, respectively. White LED (IP65 Dimmable LED Bulb, B-E27:10 W, 5 K) bulbs were used for lighting, and the lighting schedule was maintained as 16 h of light and 8 h of dark using an automatic timer throughout the study. Ambient temperature and relative humidity within the shed were measured twice daily, once in the morning and once in the afternoon, at the level of the birds using a calibrated thermometer-hygrometer (Temp Alert, model R17HE910, S4GEM35XB, FCC RoHS compliant, 2011/65/EU, Winland Electronics, Inc., Mankato, WI, USA). Weekly averages of temperature and relative humidity within the housing environment are presented in the Supplementary Materials (Figure S1a,b).

### 2.2. Experimental Design and Diets

There were eight dietary treatments, including a standard protein (SP) diet, a reduced protein (RP) diet and RP diet supplemented with two levels (0.06% or 0.12%) of either Arg, Cit or GAA, resulting in the remaining six dietary treatments. Each treatment had 13 replicate cages of two hens per cage per treatment (n = 208). Each experimental unit consisted of an individual cage housing two hens. Using previous food conversion ratio and egg mass data of hens housed in pairs in our experimental facility, we calculated that 13 replicate cages should demonstrate a significant difference of 4% or more in feed conversion efficiency with an experimental power of 0.8 and confidence level of 0.05. This study was conducted in a layer cage facility over 16 weeks from 60 to 75 weeks of age. Initial average body weights of hens did not differ significantly among dietary treatment groups (*p* > 0.05). All diets were formulated primarily using wheat, soybean meal, sorghum, barley, and canola meal. Arginine, GAA, and Cit were supplemented to the RP diet by partially substituting wheat content. The SP diet with standard CP level (15.8%) and RP diet with 2 percentage points lower CP level compared to the SP diet (13.8%) were formulated according to Hy-Line Brown nutritional recommendations [40], where amino acid balance was maintained and diets were isoenergetic. Dietary Arg, GAA and Cit supplemental levels were selected based on the manufacturer’s recommended dosages and published reports [41]. Supplemental L-Arg and L-Cit were purchased from BulkSupplements.com (https://www.bulksupplements.com/, accessed on 1 February 2023). The purity of L-Arg was 98%, while L-Cit had a purity of 98.5%, according to the manufacturer’s specifications. Guanidinoacetic acid was sourced from AlzChem (https://philagro.co.za/wp-content/uploads/2022/03/20210329_Technical-Data-Sheet_EN.pdf, accessed on 1 February 2023), with a reported purity of 96%. Feed was provided as mash. Titanium dioxide was added to all diets at 0.5% as an internal marker for the digestibility assay [42].

The nutritional profiles of the major feed ingredients, encompassing parameters such as dry matter (DM), apparent metabolizable energy (AMEn), CP, crude fat, crude fiber, AA, as well as mineral and ash contents, were analyzed using a near-infrared reflectance (NIR) spectroscopy system (Foss NIR 6500, FOSS Analytics, Hillerød, Denmark) [43] standardized with Evonik AMINONIR Advanced calibration (Evonik Industries, Essen, Germany). These results were then used for diet formulation. The diet composition and calculated nutrient values of the SP and basal RP diets are shown in Table 1. The analyzed nutrient values of the final diets, including DM, gross energy (GE), CP, ash, calcium, total phosphorus and AA, were determined using standard methods [44] and are presented in Table 2. Generally, the analyzed nutrient content of the final diets was close to the calculated values.

### 2.3. Data Collection

Data collection was conducted over a 16-week period. Eggs were collected and individually weighed on a daily basis, while feed intake was recorded weekly. Hen body weights were measured at 60 and 75 weeks of age. Egg mass and feed conversion ratio (FCR; expressed as kg of feed per kg of eggs produced) were calculated using egg production data, average egg weight, and feed consumption. Egg quality assessments were performed at 75 weeks of age. In addition, eight hens per dietary treatment were randomly selected (random selection of the replicate cage and random selection of the hen within the cage), rendered unconscious via electrical stunning (MEFE CAT 44N, Mitchell Engineering Food Equipment, Clontarf, QLD, Australia), and euthanized by decapitation for the collection of blood serum, ileal digesta, and bone samples. The collected samples were subsequently analyzed to determine serum uric acid level, ileal protein and energy digestibility, and bone quality parameters.

### 2.4. Egg Quality Analysis

A total of 97 eggs (approximately 12 eggs per treatment selected from replicates at random, except for broken eggs, which were discarded due to being unable to take accurate measurements with a maximum of one egg per replicate chosen) were collected shortly after laying at 75 weeks of age for egg quality analysis. Any significantly deformed eggs were excluded from the analysis. Egg length and width were measured using a digital Vernier caliper (Kincrome^®^, 0–150 mm range, Scoresby, VIC, Australia), and the egg shape index was calculated as the ratio of width to length. Eggshell reflectivity was determined using a shell reflectivity meter (Technical Services and Supplies, Dunnington, York, UK). Eggshell breaking strength, along with internal quality parameters, was evaluated using a digital egg tester (DET6500^®^, Nabel Co., Ltd., Kyoto, Japan). The egg yolk was separated from egg albumin using Whatman filter papers (CAT No. 1541–090, Whatman^®^, Amersham, UK) and weighed. The eggshells were washed, air-dried for at least 72 h, and weighed using a precision analytical balance (AdventurerTM, Model AX423, Ohaus, Parsippany, NJ, USA), and then the eggshell thickness (including membrane) was measured using a custom-built gauge (Mitutoyo Dial Comparator Gauge, Model 2109-10, Kawasaki, Japan). To measure egg proportions, albumen weight was calculated by subtracting yolk weight and shell weight from the intact egg weight. Percentages of albumen, yolk, and eggshell were obtained by dividing the weights of these egg components by the total egg weight. All measures were performed by trained personnel within 3 h of the egg collection.

### 2.5. Analysis of Serum Uric Acid

Blood samples were collected into Vacutainers (Becton, Dickinson U.K. Limited, Plymouth, UK) coated with silica and a polymer gel to obtain serum samples and were kept on ice immediately after collection until processing. Samples were then centrifuged at 3000× *g* at 4 °C for 10 min within 5 h of blood collection at 75 weeks of age. Following processing, serum samples were stored at −20 °C for further analysis. Serum uric acid levels were measured in duplicate using an integrated chemistry analyzer (Reference number: DF77 [URCA Uric Acid], Siemens Dimension Xpand Plus, Siemens Healthcare, Newark, NJ, USA).

### 2.6. Energy and Protein Digestibility

At 75 weeks of age, the whole ileum of each sampled hen was removed, starting at Meckel’s diverticulum and extending 1 cm anterior to the ileocecocolonic junction. The ileal content was collected by gentle squeezing into 70 mL containers, frozen at −20 °C, freeze-dried (Christ Alpha 1-4 LD plus, Martin Christ Gefriertrocknungsanlagen GmbH, Osterode am Harz, Germany), then ground using an ultra-centrifugal mill (Retsch ZM 200, Fisher Scientific, Hampton, NH, USA). A nitrogen analyzer (LECO Corporation, St Joseph, MI, US) was used to determine the nitrogen content of feed and ileal digesta samples using the Dumas combustion method with EDTA as a calibration standard [45]. Using the benzoic acid as a standard, the Parr adiabatic oxygen bomb calorimeter (Parr Instrument Co., Moline, IL, USA) was calibrated to determine the gross energy levels in feed and ileal digesta samples. The titanium content in ground diets and ileal digesta samples was determined in duplicates by using an established method [46]. Samples with a variation greater than 5% between duplicates were reanalyzed. The apparent ileal energy (IDEC) and protein (IDNC) digestibility coefficients were calculated using the following formula Jasek et al. [47].

$$IDNC=1-\left(\frac{{Ti}_{diet}\times {CP}_{digesta}}{{Ti}_{digesta}\times {CP}_{diet}}\right)$$

$$IDEC=1-\left(\frac{{Ti}_{diet}\times {GE}_{digesta}}{{Ti}_{digesta}\times {GE}_{diet}}\right)$$

where ${GE}_{diet}$ and ${GE}_{digesta}$ were gross energy of the diet and ileal digesta samples, respectively, ${Ti}_{diet}$ and ${Ti}_{digesta}$ were the titanium dioxide content in the diet and ileal digesta samples, respectively, and CP indicated either feed or ileal digesta CP content.

The DM of the feed samples and freeze-dried digesta samples was determined by drying the samples in a drying oven at 105 °C for 24 h for calculations of nutrient digestibility on a DM basis.

### 2.7. Bone Quality Parameters

Right leg bones including the tibia and femur were separated, cleaned, and air-dried in a fume hood for 48 h. Fresh and air-dried bone weights were recorded. A Vernier caliper with a 0–150 mm range (Kincrome^®^, Scoresby, Victoria, Australia) was used to measure bone length and width, and an electromechanical universal testing machine (Instron^®^ Mechanical Testing Systems, Norwood, MA, USA) was used to determine the bone breaking strength according to Dao et al. [48]. For statistical analysis, tibia and femur breaking strength data were log-transformed prior to analysis to meet assumptions of normality and homogeneity of variance.

### 2.8. Statistical Analysis

Data were analyzed using R Commander (version 3.3.1, R Foundation for Statistical Computing, Vienna, Austria). Cage was considered the experimental unit for all parameters except egg quality, in which the egg was defined as the experimental unit. Prior to statistical analyses, data were tested for normal distribution and homogeneity of variances between the dietary treatments. Depending on the results of these two tests, either one-way ANOVA or the non-parametric ANOVA (Kruskal–Wallis test) was employed to test statistical differences between the eight dietary treatments, to determine the effect of reducing dietary crude protein and compare this with the Arg, GAA and Cit inclusion to RP diets (subsequently Dunn’s Test was used for post-hoc analysis of the Kruskal–Wallis test). Additionally, a two-way ANOVA (excluding treatment 1 and 2; SP and RP control diets) was then used to test the interaction between Arg sources (Arg, GAA or Cit) and level (low (0.06%) or high (0.12%)). Tukey’s post-hoc test was subsequently used to identify pairwise differences between the treatment means. The differences were considered significant at the 5% probability level.

## 3. Results

### 3.1. Laying Performance and Hen Weight

Mortality during the experimental period was very low (<0.5%) and was not related to dietary treatment. An Arg source × level interaction was detected for feed intake during 60–67 weeks (*p* = 0.007) and 60–75 weeks (*p* = 0.014), indicating that responses differed depending on the Arg metabolic source. In these periods, increasing Cit supplementation consistently increased feed intake, whereas Arg and GAA had no effect. A similar pattern was observed from 68 to 75 weeks, although the interaction was not statistically significant (*p* = 0.055), suggesting a comparable but weaker response.

Dietary Arg source also influenced egg performance. From 60 to 67 weeks, hens fed Cit showed higher egg weight (*p* = 0.049) and a tendency for increased egg mass (*p* = 0.063) compared with GAA. From 68 to 75 weeks, Cit supplementation resulted in higher egg mass (*p* = 0.014) and feed intake (*p* = 0.009) than both Arg and GAA treatments. In addition, Cit tended to improve egg weight compared with GAA (*p* = 0.053) and showed a tendency for higher hen day egg production compared with Arg (*p* = 0.080).

Over the entire experimental period (60–75 weeks), Cit supplementation resulted in higher egg weight (*p* = 0.048) and egg mass (*p* = 0.019) compared with GAA, confirming a consistent advantage in productive output.

One-way ANOVA further confirmed these patterns, showing that hens fed RP diets supplemented with 0.12% Cit had higher feed intake (*p* < 0.001) and tended to have higher egg mass (*p* = 0.058) than those fed SP and RP diets from 60–67 weeks. Similar improvements in feed intake and egg mass were observed across 68–75 and 60–75 weeks (all *p* < 0.05), indicating a consistent response to higher Cit inclusion.

No differences were observed in FCR, final body weight at 75 weeks, or body weight gain among dietary treatments. Laying performance results are presented for 60–67, 68–75, and 60–75 weeks of age (Table 3, Table 4 and Table 5), and hen weight and weight gain are shown in Table 6. Body weight variables were included only as supportive descriptive parameters and not as target performance indicators.

### 3.2. Internal and External Egg Quality

Egg quality parameters were largely unaffected by dietary treatments at 75 weeks of age. Also, external egg quality traits did not differ among treatments.

For internal egg quality, hens fed diets supplemented with Cit tended to have higher yolk diameter than those fed GAA (*p* = 0.086). Arginine source also affected yolk weight (*p* = 0.018), with hens fed Arg and Cit having higher yolk weights (17.7 g) than those fed GAA (16.6 g) at 75 weeks. One-way ANOVA further showed that hens fed the RP diet supplemented with low-level Arg had higher yolk weight than those fed low-level GAA (18.2 vs. 16.4 g; *p* = 0.024). No differences were observed between SP and RP diets for overall egg quality traits. The external egg quality, internal egg quality, and egg proportions of hens fed the dietary treatments at 75 weeks of age are presented in Table 7, Table 8 and Table 9, respectively.

### 3.3. Serum Uric Level and Ileal Protein and Energy Digestibility Coefficient

No significant Arg source × level interactions were observed for ileal protein digestibility, ileal energy digestibility, or serum uric acid concentration.

Low-level Arg supplementation with the RP diet significantly increased ileal energy digestibility compared with the SP and RP diets (*p* = 0.037), representing the only clear effect observed for nutrient digestibility. Other comparisons for ileal energy digestibility were not significant. Also, serum uric acid concentration was not affected by Arg source or level. The results for ileal protein and energy digestibility and serum uric acid concentrations at 75 weeks of age are presented in Table 10.

### 3.4. Bone Quality Parameters

Interactions between Arg source × level were observed for tibia (*p* = 0.014) and femur (*p* = 0.006) breaking strength. In contrast to Arg and GAA, higher levels of Cit supplementation increased tibia and femur breaking strength. A main effect of Arg level was also detected, with low-level supplementation significantly increased fresh tibia weight (*p* = 0.020) and femur length (*p* = 0.038) compared with high levels.

One-way ANOVA confirmed that Cit supplementation at a high-level improved tibia and femur breaking strength, and GAA at a low-level increased tibia breaking strength compared with the RP control diet (*p* = 0.003 and *p* = 0.019, respectively). The bone quality parameters of hens fed dietary treatments at 75 weeks of age are shown in Table 11.

## 4. Discussion

Given that Arg, GAA, and Cit can all contribute to Arg-related physiological functions in poultry [23], and that Cit supplementation has been reported to increase plasma Arg concentrations and enhance NO synthesis more efficiently than Arg supplementation [49,50,51], it was hypothesized that Cit may provide greater benefits than Arg when included in laying hen diets during the late laying phase. In the present study, high-level Cit supplementation in the RP diet increased feed intake and egg mass during 60–75 weeks of age compared with both the RP and SP treatments, without affecting hen body weight. Notably, only Cit supplementation increased feed intake, whereas Arg and GAA did not affect it. A possible explanation for the overall higher feed intake observed in the present study is that birds consumed feed above strain recommendations, likely due to the colder climatic conditions during the trial, which may also represent a methodological limitation of the study. In addition, Cit consistently improved egg mass and/or egg weight relative to the Arg and GAA treatments, suggesting that Cit may exert superior effects on laying performance under the conditions of this study. The increased egg mass observed with high Cit supplements may be partly attributed to the associated increase in feed intake.

In contrast, neither low nor high levels of Arg or GAA supplementation influenced the production performance of hens fed RP diets. The lack of response to GAA supplementation observed in the present study is generally consistent with previous reports. Khakran et al. [52] found no significant effects of incremental GAA supplementation (0.057–0.171%) on feed intake, egg mass, or hen day egg production, although egg weight declined at the highest inclusion level. Similarly, Azizollahi et al. [53] reported no effects of 0.6 g/kg GAA on feed intake, egg weight, or body weight, despite improvements in egg production and egg mass. In contrast, Salah et al. [54] observed increased egg production and egg mass at 1.5 g/kg GAA in aged laying hens. These inconsistent findings may reflect differences in hen age, dietary composition, and GAA inclusion levels across studies.

Arginine is an essential AA involved in multiple metabolic processes related to poultry performance, including protein synthesis and the Arg-Cit-NO pathway [55,56,57]. Nitric oxide has been associated with reproductive and metabolic regulation and may influence follicular development, ovulation, and appetite-related pathways in poultry [58,59,60,61]. This may partially explain the increased feed intake observed in hens receiving the RP diet supplemented with high levels of Cit in the present study. Previous studies have also shown that Arg supplementation can improve egg production, egg quality, and feed efficiency in laying hens, ducks, and broiler breeder hens [29,30,62,63,64]. Several factors may explain the comparatively greater response to Cit supplementation observed in the current study. Unlike Arg, Cit is not extensively degraded by intestinal arginase, which may allow greater post-absorptive availability of Arg precursors [65]. In addition, dietary Arg undergoes substantial first-pass metabolism, potentially limiting its systemic availability [66]. In contrast, Cit is converted to Arg mainly in the kidneys through the actions of argininosuccinate synthetase and argininosuccinate lyase before entering circulation [67]. Additionally, Cit may pose a lower risk of gastrointestinal discomfort or toxicity compared with Arg [68]. These metabolic differences may contribute to the improved performance responses observed with Cit supplementation in RP diets.

Reduced CP diets supplemented with Arg precursors are an established strategy to reduce feed costs while maintaining laying hen performance [69,70]. A recent study by Iqbal et al. [71] reported that low protein diets reduced feed costs by approximately AUD 8.50 per ton, indicating potential economic benefits for the poultry industry. Chrystal et al. [72] further highlighted that RP diet development enhances sustainability through more efficient nutrient use and reduced reliance on soybean meal. In the present study, AA supplementation enabled a reduction of approximately 40% in soybean meal inclusion, demonstrating a practical formulation advantage of RP diets in reducing dependence on conventional protein sources. Importantly, despite this substantial reduction in CP, productive performance was maintained when diets were balanced with essential AA. This is consistent with Ji et al. [73], who reported no adverse effects on egg production or FCR in hens fed low protein diets during early lay. Similarly, Keshavarz and Austic [74] showed that egg production was maintained in hens fed 13% CP diets supplemented with essential AA compared with 16% CP diets. Dao et al. [16] also demonstrated that RP diets supplemented with Arg metabolites sustained egg production comparable to higher protein diets during early laying.

However, responses to RP diets may depend on physiological stage and dietary balance. Novak et al. [75] reported that older hens may show greater sensitivity to CP reduction, with potential declines in performance when AA supply is limiting. In contrast, the present study demonstrates that a moderate reduction in CP (approximately two percentage points), when balanced with essential AA, is sufficient to sustain productive performance in aged laying hens (60–75 weeks). This highlights the specific contribution of this study in confirming that late-phase hens can maintain performance under RP feeding when diets are properly balanced. Furthermore, a key finding of this study is that high-level Cit supplementation further increased egg mass compared with both RP and SP treatments. This indicates that Cit may act as a more effective functional Arg precursor under RP conditions, providing additional productivity benefits beyond AA balancing alone.

The results of this study showed that Arg, GAA, and Cit supplementation to RP diets did not affect external or most internal egg quality parameters. These findings are consistent with Dao et al. [16], who reported no effects of Arg, GAA, or Cit supplementation on shell strength, shell reflectivity, albumen height, yolk color, or Haugh unit in laying hens. Similarly, previous studies have shown that egg quality parameters remain largely unaffected by moderate reductions in dietary CP (15.5–17.5%) [75,76]. However, a notable finding of the present study was that RP diets with Arg and Cit supplementation increased yolk weight compared with RP-GAA supplementation. This is consistent with Al-Bayar et al. [77], who observed increased yolk weight with Arg supplementation in laying turkeys, and Xia et al. [29], who reported improved egg yolk percentage in laying ducks with appropriate Arg levels (1.26%). The improved yolk response in the RP-Arg and RP-Cit groups may be associated with enhanced Arg availability and related metabolic functions, whereas GAA primarily supports energy metabolism without directly influencing reproductive hormone pathways. These differences may contribute to the observed variation in yolk development among treatments. However, responses to GAA supplementation have been inconsistent across studies, with some reporting improvements in egg quality traits [54] and others showing no effects [16]. These discrepancies are likely due to differences in bird age, genetic background, and dietary formulation.

In the present study, supplementation of the RP diet with a low level of Arg significantly increased ileal energy digestibility compared with both RP and SP diets at 75 weeks of age. This suggests that an optimal level of dietary Arg may enhance energy utilization in aged laying hens, potentially through improved intestinal function and nutrient absorption. Previous studies have proposed that Arg supports gut health and nutrient uptake via nitric oxide-mediated pathways and polyamine synthesis [78,79]. In contrast, higher supplementation did not provide additional benefits for energy digestibility in the current study. Additionally, RP diets supplemented with a low level of Cit showed a trend toward reduced serum uric acid levels compared with the SP diet at 75 weeks of age. Serum uric acid is commonly used as an indirect marker of protein utilization and nitrogen metabolism [80]. This trend may suggest improved protein utilization efficiency under RP feeding with Cit supplementation. Consistent with previous reports, higher dietary CP has been associated with increased serum uric acid due to greater AA catabolism [16,81]. However, in the present study, this response did not reach statistical significance and should therefore be interpreted with caution.

Arginine has been reported to contribute to bone development through its role in collagen synthesis and connective tissue formation [82,83]. In this study, high-level Cit supplement in the RP diet was associated with improved bone breaking strength in aged laying hens. This finding suggests a potential beneficial effect of Cit on skeletal integrity during the late laying phase. A possible explanation may involve enhanced Arg availability through Cit metabolism, which supports NO production. Nitric oxide has been proposed to regulate bone remodeling by suppressing osteoclast activity (bone resorption) and promoting osteoblast (bone-forming cells) function, thereby contributing to bone formation and mineralization [84]. Similarly, a study found that daily oral Cit supplementation at 5 g/kg body weight increased the concentrations of Cit and ornithine in wild mice after 14 days, possibly indicating the development of collagen synthesis through the production of polyamine, hence improving the bone support [85], which is in agreement with our data. Improved bone strength may have important implications for hen welfare and may help reduce osteoporosis-related mortality in aged laying hens [86], potentially supporting longer productive laying cycles, while reducing their economic losses.

In contrast, high-level supplementation of Arg or GAA did not confer similar benefits as high-Cit level, when compared to their respective low-level inclusions. Khajali et al. [35] reported that supplementing broiler chicks with low levels of GAA at 0.06% increased tibia density and breaking strength up to 24 days of age, which agrees with our findings that a low dose of GAA supplementation (0.06%) increased bone breaking strength compared to higher levels at 75 weeks of age. Fernandes et al. [87] described that the femur and tibia bone quality parameters were not significantly different at various dietary Arg levels (1.30, 1.45, 1.60, 1.75 and 1.90%) on days 7 or 21. However, femur length, tibia diameter, and the seedor index of both bones increased linearly in broiler breeders fed the Arg diet on day 7, and only the femur index seedor was improved on day 21. Another study by Castro et al. [88] stated that bone mineral density increased with increasing Arg concentration at 42 days of age. However, the bone growth rate was not affected by varying Arg levels (70, 80, 90, 100, and 110%). Additionally, mRNA expressions of key genes such as nitric oxide synthase 2 (NOS2) and peroxisome proliferator-activated receptor gamma (PPAR-γ), which are essential for bone remodeling and density, remained unchanged despite higher Arg level [88]. This can be explained by differences in growth phases and bone remodeling capacities between young and old birds. Hence, supplementing high amounts of Arg leads to increased bone diameter in young birds due to active bone growth and remodeling, but less influence on bone strength in older birds due to slower remodeling and mature bones. This idea aligns with the study of Agarwal et al. [89], who recorded an interesting finding that when male mice were fed Arg and Cit treatments at various doses of 2.5, 7.5, and 12.5 g/kg, Cit supplements bypassed the first pass metabolism and increased Arg flow by 86%, leading to a significant rise in plasma Arg levels. However, 70% of the supplemented Arg was absorbed by organs and disappeared before reaching the bloodstream, with only 30% entering the peripheral circulation after 2 weeks of study. This highlights how splanchnic organs, like the liver, extensively limit the availability of Arg supplements despite higher concentrations. Cit supplementation can, therefore, lead to better bone matrix synthesis, bone mineralization, and osteoblast function in older laying hens than Arg.

## 5. Conclusions

In conclusion, a moderate reduction in dietary CP (two percentage points), when supplemented with Arg precursors, was effective in maintaining productive performance in aged laying hens. Among the supplements evaluated, high-level Cit provided the greatest overall benefits by improving egg mass, egg weight, yolk weight, and bone breaking strength, potentially through enhanced Arg-related metabolic pathways. In addition, low-level Arg supplements improved ileal energy digestibility, while low-dose GAA supplements improved bone breaking strength. Moreover, hens fed Arg and Cit diets had higher yolk weights. These findings suggest that RP feeding strategies supplemented with functional Arg precursors may support productive efficiency, skeletal health, and nutrient utilization of late-lay hens. Also, this approach has potential economic and environmental benefits by reducing reliance on soybean meal, which may decrease feed cost and improve nutrient efficiency.

## Figures and Tables

**Table 1:** Diet composition and calculated nutrient values of basal standard and reduced protein diets (as-fed basis).

Ingredients, %	Standard Protein Diet	Reduced Protein Diet
Wheat	32.4	33.8
Sorghum	27.5	29.5
Soybean meal	15.6	9.4
Barley	10.0	12.5
Canola meal	2.3	2.5
Limestone grit	6.8	6.8
Fine limestone	3.0	3
Canola Oil	1.10	0.60
Titanium dioxide	0.5	0.5
Salt	0.15	0.090
Sodium bicarbonate	0.14	0.240
D,L-methionine	0.125	0.185
Monocalcium phosphate	0.1	0.2
Choline chloride 60%	0.09	0.115
L-lysine HCl	0.045	0.23
Xylanase ^1^	0.01	0.01
Phytase ^2^	0.006	0.006
Layer vitamin-mineral premix ^3^	0.1	0.1
Pigment Jabiru red	0.004	0.004
Pigment Jabiru yellow	0.003	0.003
L-valine	0.00	0.03
L-isoleucine	0.00	0.04
L-threonine	0.00	0.05
Potassium carbonate	0.00	0.05
Total	1000	1000
Calculated nutrient, % (otherwise as indicated)		
AME ^4^, kcal/kg	2690	2690
Crude protein	15.8	13.8
Crude fat	3.00	2.60
Crude fibre	2.80	2.80
Ash	13.1	13.0
Total lysine	0.753	0.734
Total methionine	0.365	0.392
Total methionine + cysteine	0.642	0.64
Total threonine	0.574	0.526
Total isoleucine	0.627	0.559
Total leucine	1.294	1.122
Total tryptophan	0.216	0.183
Total arginine	0.927	0.747
Total histidine	0.375	0.315
Total valine	0.731	0.653
Total glycine	0.614	0.516
Total phenylalanine	0.768	0.651
Digestible ^5^ lysine	0.670	0.670
Digestible methionine	0.359	0.386
Digestible methionine + cysteine	0.603	0.603
Digestible threonine	0.507	0.469
Digestible isoleucine	0.583	0.523
Digestible leucine	1.206	1.045
Digestible tryptophan	0.195	0.165
Digestible arginine	0.876	0.704
Digestible histidine	0.343	0.287
Digestible valine	0.659	0.590
Digestible glycine	0.555	0.464
Digestible phenylalanine	0.708	0.599
Calcium	4.000	4.000
Available phosphorus	0.332	0.32
Total phosphorus	0.352	0.343
Sodium	0.160	0.160
Chloride	0.161	0.161
Potassium	0.602	0.540
Choline, mg/kg	1600	1600
Linoleic acid	1.200	1.100
Dietary electrolyte balance ^6^, mEq	178	162

^1^ Axtra^®^ XB 201 TPT, Danisco Animal Nutrition, IFF, Marlborough, UK; ^2^ Axtra^®^ PHY Gold 10,000 FTU/g, Danisco Animal Nutrition, IFF, UK; ^3^ Vitamin-mineral premix (Rabar Pty Ltd., Beaudesert, Queensland, Australia) provided the following per kilogram diet: vitamin A, 10,000 IU; vitamin D, 3000 IU; vitamin E, 20 mg; vitamin K, 3 mg; nicotinic acid (niacin), 35 mg; pantothenic acid, 12 mg; folic acid, 1 mg; riboflavin (B2), 6 mg; cyanocobalamin (B12), 0.02 mg; biotin, 0.1 mg; pyridoxine (B6), 5 mg; thiamine (B1), 2 mg; copper, 8 mg as copper sulphate pentahydrate; cobalt, 0.2 mg as cobalt sulphate 21%; molybdenum, 0.5 mg as sodium molybdate; iodine, 1 mg as potassium iodide 68%; selenium, 0.3 mg as selenium 2%; iron, 60 mg as iron sulphate 30%; zinc, 60 mg as zinc sulphate 35%; manganese, 90 mg as manganous oxide 60%; antioxidant, 20 mg; ^4^ AMEn: N corrected apparent metabolizable energy; ^5^ Digestible amino acid coefficients of conventional feed ingredients were determined by near-infrared spectroscopy (Foss NIR 6500, Hillerød, Denmark) standardized with Adisseo calibration; ^6^ Dietary electrolyte balance was calculated as 10,000 × (Na^+^ + K^+^ − Cl^−^).

**Table 2:** Analyzed nutrient content of the dietary treatments (as-fed basis, % otherwise as stated) ^1^_._

Nutrient Composition	SP	RP	RP-Arg Low	RP-Arg High	RP-Cit Low	RP-Cit High	RP-GAA Low	RP-GAA High
Dry matter	90.4	90.3	90.7	90.6	90.6	90.6	90.7	91.1
Gross energy (Kcal/kg)	3583	3569	3568	3571	3569	3565	3616	3478
Crude protein	15.1	13.4	13.4	13.7	13.6	13.8	14.0	13.3
Ash content	12.6	12.2	12.9	12.3	12.6	12.6	11.6	14.5
Calcium	3.35	3.16	3.63	3.18	3.43	3.81	2.96	3.52
Total phosphorus	0.33	0.31	0.33	0.33	0.34	0.35	0.36	0.36
Arginine	7.90	5.31	7.67	8.20	6.70	7.06	6.84	6.73
Methionine	3.51	3.32	3.32	3.32	3.32	3.32	3.32	3.32
Lysine	6.47	6.00	6.00	6.00	6.00	6.00	6.00	6.00
Threonine	4.61	3.58	3.58	3.58	3.58	3.58	3.58	3.58
Valine	5.03	4.00	4.00	4.00	4.00	4.00	4.00	4.00
Isoleucine	5.24	4.19	4.19	4.19	4.19	4.19	4.19	4.19
Leucine	11.3	8.65	8.65	8.65	8.65	8.65	8.65	8.65
Phenylalanine	7.53	5.30	5.30	5.30	5.30	5.30	5.30	5.30
Histidine	3.10	2.17	2.17	2.17	2.17	2.17	2.17	2.17
Serine	6.93	5.05	5.05	5.05	5.05	5.05	5.05	5.05
Glycine	6.37	4.49	4.49	4.49	4.49	4.49	4.49	4.49
Alanine	6.19	4.73	4.73	4.73	4.73	4.73	4.73	4.73
Proline	10.2	8.09	8.09	8.09	8.09	8.09	8.09	8.09
Glutamic Acid	27.3	21.2	21.2	21.2	21.2	21.2	21.2	21.2
Tyrosine	4.02	2.97	2.97	2.97	2.97	2.97	2.97	2.97
Aspartic acid	10.3	6.96	6.96	6.96	6.96	6.96	6.96	6.96

^1^ Values of all the amino acids presented were total amino acids (measured on an as-is basis).

**Table 3:** Laying performance of hens fed the dietary treatments from weeks 60 to 67.

Effects		Egg Weight (g)	Hen Day Egg Production (%)	Egg Mass (g)	Feed Intake (g)	FCR (kg Feed/kg Egg)
Two-way ANOVA results (2 × 3 factorial arrangement of treatments)						
Treatment	RP-Arg low	64.3	95.5	61.3	136 ^ab^	2.235
	RP-Arg high	65.0	95.3	62.0	133 ^a^	2.155
	RP-Cit low	65.1	94.7	61.7	134 ^a^	2.184
	RP-Cit high	66.2	97.7	65.0	142 ^b^	2.203
	RP-GAA low	63.3	95.8	60.9	130 ^a^	2.143
	RP-GAA high	63.5	95.8	60.8	128 ^a^	2.122
Source	Arg	64.7 ^ab^	95.4	61.7	134 ^b^	2.193
	Cit	65.7 ^b^	96.3	63.4	138 ^b^	2.194
	GAA	63.4 ^a^	95.8	60.9	129 ^a^	2.133
Level	Low	64.2	95.4	61.3	133	2.187
	High	64.9	96.2	62.5	134	2.159
SEM		0.37	0.41	0.44	0.91	0.015
*p*-values	Source	0.049	0.673	0.063	<0.001	0.144
	Level	0.383	0.333	0.183	0.600	0.307
	Source × level	0.857	0.222	0.270	0.007	0.388
One-way ANOVA results						
Treatment	SP	63.5	95.2	60.2	133 ^a^	2.211
	RP	63.3	94.8	60.0	129 ^a^	2.157
	RP-Arg low	64.3	95.5	61.3	136 ^ab^	2.235
	RP-Arg high	65.0	95.3	62.0	133 ^a^	2.155
	RP-Cit low	65.1	94.7	61.7	134 ^ab^	2.184
	RP-Cit high	66.2	97.7	65.0	142 ^b^	2.203
	RP-GAA low	63.3	95.8	60.9	130 ^a^	2.143
	RP-GAA high	63.5	95.8	60.8	128 ^a^	2.122
SEM		0.31	0.38	0.39	0.82	0.012
*p*-value		0.167	0.668	0.058	<0.001	0.248

^a,b^ Means within columns not sharing a common suffix are significantly different at the 5% level of probability. SP: A standard protein diet contains 15.8% crude protein. RP: A reduced protein diet contains 13.8% crude protein. RP-Arg low, RP-Cit low, RP-GAA low: RP diet supplemented with Arg, Cit and GAA at the low level (0.06% or 0.6 g/kg), respectively. RP-Arg high, RP-Cit high, RP-GAA high: RP diet supplemented with Arg, Cit and GAA at the high level (0.12% or 1.2 g/kg), respectively.

**Table 4:** Laying performance of hens fed the dietary treatments from weeks 68 to 75.

Effects		Egg Weight (g)	Hen Day Egg Production (%)	Egg Mass (g)	Feed Intake (g)	FCR (kg Feed/kg Egg)
Two-way ANOVA results (2 × 3 factorial arrangement of treatments)						
Treatment	RP-Arg low	64.4	94.0	60.5	127	2.117
	RP-Arg high	64.9	93.8	61.0	125	2.070
	RP-Cit low	65.4	96.3	63.0	127	2.030
	RP-Cit high	66.6	97.0	64.8	136	2.109
	RP-GAA low	63.9	94.0	60.1	125	2.087
	RP-GAA high	63.5	95.4	60.8	125	2.075
Source	Arg	64.6	93.9	60.8 ^a^	126 ^a^	2.092
	Cit	66.0	96.6	63.9 ^b^	132 ^b^	2.071
	GAA	63.7	94.7	60.4 ^a^	125 ^a^	2.081
Level	Low	64.6	94.7	61.1	126	2.081
	High	65.0	95.3	62.1	128	2.084
SEM		0.39	0.50	0.52	0.98	0.016
*p*-values	Source	0.053	0.080	0.014	0.009	0.864
	Level	0.546	0.525	0.351	0.310	0.922
	Source × level	0.731	0.815	0.851	0.055	0.274
One-way ANOVA results						
Treatment	SP	63.5	92.9	58.8 ^a^	125 ^a^	2.130
	RP	63.2	92.9	58.8 ^a^	123 ^a^	2.113
	RP-Arg low	64.4	94.0	60.5 ^ab^	127 ^ab^	2.117
	RP-Arg high	64.9	93.8	61.0 ^ab^	125 ^a^	2.070
	RP-Cit low	65.4	96.3	63.0 ^ab^	127 ^ab^	2.030
	RP-Cit high	66.6	97.0	64.8 ^b^	136 ^b^	2.109
	RP-GAA low	63.9	94.0	60.1 ^ab^	125 ^a^	2.087
	RP-GAA high	63.5	95.4	60.8 ^ab^	125 ^a^	2.075
SEM		0.33	0.49	0.48	0.86	0.013
*p*-value		0.141	0.339	0.030	0.008	0.688

^a,b^ Means within columns not sharing a common suffix are significantly different at the 5% level of probability. SP: A standard protein diet contains 15.8% crude protein. RP: A reduced protein diet contains 13.8% crude protein. RP-Arg low, RP-Cit low, RP-GAA low: RP diet supplemented with Arg, Cit and GAA at the low level (0.06% or 0.6 g/kg), respectively. RP-Arg high, RP-Cit high, RP-GAA high: RP diet supplemented with Arg, Cit and GAA at the high level (0.12% or 1.2 g/kg), respectively.

**Table 5:** Laying performance of hens fed the dietary treatments from weeks 60 to 75.

Effects		Egg Weight (g)	Hen Day Egg Production (%)	Egg Mass (g)	Feed Intake (g)	FCR (kg Feed/kg Egg)
Two-way ANOVA results (2 × 3 factorial arrangement of treatments)						
Treatment	RP-Arg low	64.3	94.8	60.9	132 ^ab^	2.176
	RP-Arg high	64.9	94.5	61.5	129 ^a^	2.113
	RP-Cit low	65.3	95.5	62.3	131 ^a^	2.107
	RP-Cit high	66.4	97.4	64.9	139 ^b^	2.156
	RP-GAA low	63.6	94.9	60.5	127 ^a^	2.115
	RP-GAA high	63.5	95.6	60.8	127 ^a^	2.099
Source	Arg	64.6 ^ab^	94.6	61.2 ^ab^	130 ^ab^	2.143
	Cit	65.8 ^b^	96.5	63.7 ^b^	135 ^b^	2.132
	GAA	63.5 ^a^	95.3	60.7 ^a^	127 ^a^	2.107
Level	Low	64.4	95.0	61.2	130	2.134
	High	65.0	95.8	62.3	131	2.121
SEM		0.38	0.39	0.46	0.90	0.014
*p*-values	Source	0.048	0.168	0.019	<0.001	0.542
	Level	0.456	0.361	0.234	0.400	0.631
	Source × level	0.790	0.557	0.543	0.014	0.275
One-way ANOVA results						
Treatment	SP	63.5	94.3	59.6 ^a^	129 ^a^	2.180
	RP	63.3	93.9	59.4 ^a^	126 ^a^	2.135
	RP-Arg low	64.3	94.8	60.9 ^ab^	132 ^ab^	2.176
	RP-Arg high	64.9	94.5	61.5 ^ab^	129 ^a^	2.113
	RP-Cit low	65.3	95.5	62.3 ^ab^	131 ^ab^	2.107
	RP-Cit high	66.4	97.4	64.9 ^b^	139 ^b^	2.156
	RP-GAA low	63.6	94.9	60.5 ^ab^	127 ^a^	2.115
	RP-GAA high	63.5	95.6	60.8 ^ab^	127 ^a^	2.099
SEM		0.32	0.40	0.42	0.80	0.012
*p*-value		0.144	0.511	0.034	<0.001	0.482

^a,b^ Means within columns not sharing a common suffix are significantly different at the 5% level of probability. SP: A standard protein diet contains 15.8% crude protein. RP: A reduced protein diet contains 13.8% crude protein. RP-Arg low, RP-Cit low, RP-GAA low: RP diet supplemented with Arg, Cit and GAA at the low level (0.06% or 0.6 g/kg), respectively. RP-Arg high, RP-Cit high, RP-GAA high: RP diet supplemented with Arg, Cit and GAA at the high level (0.12% or 1.2 g/kg), respectively.

**Table 6:** Hen weight and weight gain of the dietary treatments.

Effects		Hen Weight Week 60 (g)	Hen Weight Week 75 (g)	Weight Gain Weeks 60–75 (g)
Two-way ANOVA results (2 × 3 factorial arrangement of treatments)				
Treatment	RP-Arg low	2059	2295	236
	RP-Arg high	2098	2305	207
	RP-Cit low	2077	2254	200
	RP-Cit high	2080	2344	264
	RP-GAA low	2029	2223	194
	RP-GAA high	2114	2292	178
Source	Arg	2079	2300	221
	Cit	2079	2303	235
	GAA	2072	2258	186
Level	Low	2055	2258	211
	High	2097	2314	216
SEM		14.26	17.30	10.99
*p*-values	Source	0.975	0.509	0.174
	Level	0.143	0.115	0.837
	Source × level	0.511	0.617	0.184
One-way ANOVA results				
Treatment	SP	2073	2305	232
	RP	2072	2286	214
	RP-Arg low	2059	2295	236
	RP-Arg high	2098	2305	207
	RP-Cit low	2077	2254	200
	RP-Cit high	2080	2344	264
	RP-GAA low	2029	2223	194
	RP-GAA high	2114	2292	178
SEM		13.00	15.24	8.96
*p*-value		0.863	0.680	0.314

SP: A standard protein diet contains 15.8% crude protein. RP: A reduced protein diet contains 13.8% crude protein. RP-Arg low, RP-Cit low, RP-GAA low: RP diet supplemented with Arg, Cit and GAA at the low level (0.06% or 0.6 g/kg), respectively. RP-Arg high, RP-Cit high, RP-GAA high: RP diet supplemented with Arg, Cit and GAA at the high level (0.12% or 1.2 g/kg), respectively.

**Table 7:** External egg quality of hens fed the dietary treatments at week 75.

Effects		Shell Breaking Strength (Kgf)	Shell Thickness (mm)	Egg Length (mm)	Egg Width (mm)	Egg Shape Index	Reflectivity (%)
Two-way ANOVA results (2 × 3 factorial arrangement of treatments)							
Treatment	RP-Arg low	3.68	0.432	58.4	45.4	0.778	28.5
	RP-Arg high	3.66	0.435	59.1	45.2	0.766	28.6
	RP-Cit low	3.16	0.422	58.5	45.0	0.770	27.8
	RP-Cit high	3.72	0.428	58.0	45.0	0.777	27.5
	RP-GAA low	3.83	0.426	57.3	44.7	0.781	28.7
	RP-GAA high	3.74	0.435	57.8	44.7	0.773	26.2
Source	Arg	3.67	0.434	58.8	45.3	0.772	28.5
	Cit	3.47	0.425	58.2	45.0	0.774	27.6
	GAA	3.78	0.431	57.6	44.7	0.777	27.4
Level	Low	3.58	0.427	58.1	45.1	0.777	28.4
	High	3.71	0.433	58.3	45.0	0.772	27.4
SEM		0.11	0.003	0.25	0.16	0.003	0.49
*p*-values	Source	0.477	0.431	0.165	0.338	0.849	0.648
	Level	0.521	0.284	0.589	0.812	0.516	0.350
	Source × level	0.412	0.931	0.630	0.903	0.515	0.512
One-way ANOVA results							
Treatment	SP	3.63	0.418	57.7	44.3	0.767	26.1
	RP	2.97	0.430	59.4	44.7	0.755	27.3
	RP-Arg low	3.68	0.432	58.4	45.4	0.778	28.5
	RP-Arg high	3.66	0.435	59.1	45.2	0.766	28.6
	RP-Cit low	3.16	0.422	58.5	45.0	0.770	27.8
	RP-Cit high	3.72	0.428	58.0	45.0	0.777	27.5
	RP-GAA low	3.83	0.426	57.3	44.7	0.781	28.7
	RP-GAA high	3.74	0.435	57.8	44.7	0.773	26.2
SEM		0.10	0.003	0.22	0.14	0.003	0.41
*p*-value		0.338	0.681	0.284	0.495	0.521	0.594

SP: A standard protein diet contains 15.8% crude protein. RP: A reduced protein diet contains 13.8% crude protein. RP-Arg low, RP-Cit low, RP-GAA low: RP diet supplemented with Arg, Cit and GAA at the low level (0.06% or 0.6 g/kg), respectively. RP-Arg high, RP-Cit high, RP-GAA high: RP diet supplemented with Arg, Cit and GAA at the high level (0.12% or 1.2 g/kg), respectively.

**Table 8:** Internal egg quality of hens fed the dietary treatments at week 75.

Effects		Albumen Height (mm)	Yolk Color	Haugh Unit	Yolk Height (mm)	Yolk Diameter (mm)	Yolk Index
Two-way ANOVA results (2 × 3 factorial arrangement of treatments)							
Treatment	RP-Arg low	7.92	12.0	86.0	21.9	44.5	0.496
	RP-Arg high	7.67	12.9	83.7	21.5	42.4	0.520
	RP-Cit low	7.68	12.7	84.4	21.3	43.2	0.495
	RP-Cit high	7.85	12.5	85.7	21.7	44.2	0.492
	RP-GAA low	8.99	12.3	93.0	21.1	42.1	0.503
	RP-GAA high	7.86	12.5	85.8	21.0	42.3	0.489
Source	Arg	7.79	12.5	84.9	21.7	43.4	0.508
	Cit	7.78	12.6	85.2	21.5	43.8	0.494
	GAA	8.40	12.4	89.2	21.0	42.2	0.496
Level	Low	8.23	12.3	88.0	21.4	43.3	0.498
	High	7.80	12.6	85.1	21.4	43.0	0.499
SEM		0.22	0.17	1.47	0.14	0.31	0.004
*p*-values	Source	0.439	0.961	0.402	0.151	0.086	0.332
	Level	0.355	0.371	0.331	0.857	0.604	0.874
	Source × level	0.490	0.381	0.498	0.589	0.105	0.175
One-way ANOVA results							
Treatment	SP	8.49	12.9	90.7	20.9	43.1	0.487
	RP	7.78	12.5	85.1	21.6	43.0	0.502
	RP-Arg low	7.92	12.0	86.0	21.9	44.5	0.496
	RP-Arg high	7.67	12.9	83.7	21.5	42.4	0.520
	RP-Cit low	7.68	12.7	84.4	21.3	43.2	0.495
	RP-Cit high	7.85	12.5	85.7	21.7	44.2	0.492
	RP-GAA low	8.99	12.3	93.0	21.1	42.1	0.503
	RP-GAA high	7.86	12.5	85.8	21.0	42.3	0.489
SEM		0.19	0.14	1.23	0.12	0.26	0.003
*p*-value		0.640	0.734	0.528	0.280	0.196	0.393

SP: A standard protein diet contains 15.8% crude protein. RP: A reduced protein diet contains 13.8% crude protein. RP-Arg low, RP-Cit low, RP-GAA low: RP diet supplemented with Arg, Cit and GAA at the low level (0.06% or 0.6 g/kg), respectively. RP-Arg high, RP-Cit high, RP-GAA high: RP diet supplemented with Arg, Cit and GAA at the high level (0.12% or 1.2 g/kg), respectively.

**Table 9:** Egg proportions of hens fed the dietary treatments at week 75.

Effects		Yolk Weight (g)	Albumen Weight (g)	Shell Weight (g)	Yolk (%)	Albumen (%)	Shell (%)
Two-way ANOVA results (2 × 3 factorial arrangement of treatments)							
Treatment	RP-Arg low	18.2	42.2	6.40	27.3	63.1	9.61
	RP-Arg high	17.1	43.6	6.51	26.1	64.3	9.62
	RP-Cit low	18.0	42.7	6.14	26.9	63.9	9.19
	RP-Cit high	17.6	41.5	6.31	26.9	63.4	9.66
	RP-GAA low	16.4	41.6	6.17	25.5	64.8	9.63
	RP-GAA high	16.7	41.4	6.35	26.0	64.2	9.85
Source	Arg	17.7 ^b^	42.9	6.45	26.7	63.7	9.61
	Cit	17.7 ^b^	42.0	6.23	26.9	63.6	9.46
	GAA	16.6 ^a^	41.5	6.26	25.8	64.5	9.74
Level	Low	17.5	42.1	6.25	26.5	64.0	9.49
	High	17.1	42.1	6.38	26.3	63.9	9.71
SEM		0.20	0.43	0.07	0.25	0.25	0.07
*p*-values	Source	0.018	0.407	0.309	0.129	0.302	0.243
	Level	0.294	0.981	0.268	0.644	0.991	0.118
	Source × level	0.277	0.487	0.970	0.390	0.280	0.442
One-way ANOVA results							
Treatment	SP	16.5 ^ab^	40.8	5.99	26.1	64.4	9.49
	RP	16.8 ^ab^	42.7	6.19	25.6	65.0	9.41
	RP-Arg low	18.2 ^b^	42.2	6.40	27.3	63.1	9.61
	RP-Arg high	17.1 ^ab^	43.6	6.51	26.1	64.3	9.62
	RP-Cit low	18.0 ^ab^	42.7	6.14	26.9	63.9	9.19
	RP-Cit high	17.6 ^ab^	41.5	6.31	26.9	63.4	9.66
	RP-GAA low	16.4 ^a^	41.6	6.17	25.5	64.8	9.63
	RP-GAA high	16.7 ^ab^	41.4	6.35	26.0	64.2	9.85
SEM		0.16	0.35	0.06	0.20	0.21	0.06
*p*-value		0.024	0.540	0.356	0.263	0.319	0.398

^a,b^ Means within columns not sharing a common suffix are significantly different at the 5% level of probability. SP: A standard protein diet contains 15.8% crude protein. RP: A reduced protein diet contains 13.8% crude protein. RP-Arg low, RP-Cit low, RP-GAA low: RP diet supplemented with Arg, Cit and GAA at the low level (0.06% or 0.6 g/kg), respectively. RP-Arg high, RP-Cit high, RP-GAA high: RP diet supplemented with Arg, Cit and GAA at the high level (0.12% or 1.2 g/kg), respectively.

**Table 10:** Ileal protein and energy digestibility and serum uric level of hens fed the dietary treatments at week 75.

Effects		Ileal Protein Digestibility Coefficient	Ileal Energy Digestibility Coefficient	Serum Uric Acid Level (mg/dL)
Two-way ANOVA results (2 × 3 factorial arrangement of treatments)				
Treatment	RP-Arg low	0.828	0.761	5.04
	RP-Arg high	0.802	0.737	5.07
	RP-Cit low	0.819	0.751	4.75
	RP-Cit high	0.813	0.726	5.43
	RP-GAA low	0.815	0.746	5.19
	RP-GAA high	0.819	0.723	5.00
Source	Arg	0.815	0.749	5.05
	Cit	0.816	0.737	5.09
	GAA	0.817	0.734	5.09
Level	Low	0.821	0.753	4.99
	High	0.811	0.729	5.17
SEM		0.004	0.007	0.09
*p*-values	Source	0.986	0.647	0.990
	Level	0.318	0.083	0.308
	Source × level	0.418	0.999	0.130
One-way ANOVA results				
Treatment	SP	0.794	0.698 ^a^	5.77
	RP	0.798	0.698 ^a^	5.19
	RP-Arg low	0.828	0.761 ^b^	5.04
	RP-Arg high	0.802	0.737 ^ab^	5.07
	RP-Cit low	0.819	0.751 ^ab^	4.75
	RP-Cit high	0.813	0.726 ^ab^	5.43
	RP-GAA low	0.815	0.746 ^ab^	5.19
	RP-GAA high	0.819	0.723 ^ab^	5.00
SEM		0.004	0.006	0.09
*p*-value		0.367	0.037	0.091

^a,b^ Means within columns not sharing a common suffix are significantly different at the 5% level of probability. SP: A standard protein diet contains 15.8% crude protein. RP: A reduced protein diet contains 13.8% crude protein. RP-Arg low, RP-Cit low, RP-GAA low: RP diet supplemented with Arg, Cit and GAA at the low level (0.06% or 0.6 g/kg), respectively. RP-Arg high, RP-Cit high, RP-GAA high: RP diet supplemented with Arg, Cit and GAA at the high level (0.12% or 1.2 g/kg), respectively.

**Table 11:** Bone quality parameters of hens fed the dietary treatments at week 75.

Effects		Tibia				Femur			
		Breaking Strength (N)	Weight of Fresh Bone (g)	Length (mm)	Width (mm)	Breaking Strength (N)	Weight of Fresh Bone (g)	Length (mm)	Width (mm)
Two-way ANOVA results (2 × 3 factorial arrangement of treatments)									
Treatment	RP-Arg low	228 ^b^	13.1	126	7.83	269 ^bc^	11.7	89.2	8.97
	RP-Arg high	180 ^a^	12.7	124	7.87	206 ^a^	11.0	88.6	8.94
	RP-Cit low	196 ^a^	12.8	125	7.85	217 ^a^	11.3	89.7	8.84
	RP-Cit high	233 ^bc^	12.7	124	7.80	290 ^c^	11.1	88.1	8.86
	RP-GAA low	261 ^c^	13.7	125	7.81	286 ^c^	11.6	89.2	8.94
	RP-GAA high	207 ^ab^	12.0	123	7.55	243 ^b^	10.3	87.4	8.64
Source	Arg	204	12.9	125	7.85	237	11.3	88.9	8.95
	Cit	214	12.7	124	7.83	253	11.2	88.9	8.85
	GAA	234	12.9	124	7.68	265	11.0	88.3	8.79
Level	Low	228	13.2 ^b^	125	7.83	257	11.5	89.4 ^b^	8.92
	High	207	12.5 ^a^	124	7.74	246	10.8	88.0 ^a^	8.81
SEM		8.45	0.17	0.46	0.04	10.58	0.18	0.32	0.07
*p*-values	Source	0.211	0.900	0.647	0.185	0.380	0.650	0.687	0.648
	Level	0.296	0.020	0.122	0.270	0.880	0.057	0.038	0.462
	Source × level	0.014	0.073	0.899	0.305	0.006	0.435	0.747	0.622
One-way ANOVA results									
Treatment	SP	222 ^ab^	13.4	126	8.07	265 ^ab^	11.4	90.5	8.96
	RP	162 ^a^	12.9	124	8.01	202 ^a^	11.2	88.6	8.95
	RP-Arg low	228 ^ab^	13.1	126	7.83	269 ^ab^	11.7	89.2	8.97
	RP-Arg high	180 ^ab^	12.7	124	7.87	206 ^ab^	11.0	88.6	8.94
	RP-Cit low	196 ^ab^	12.8	125	7.85	217 ^ab^	11.3	89.7	8.84
	RP-Cit high	233 ^b^	12.7	124	7.80	290 ^b^	11.1	88.1	8.86
	RP-GAA low	261 ^b^	13.7	125	7.81	286 ^ab^	11.6	89.2	8.94
	RP-GAA high	207 ^ab^	12.0	123	7.55	243 ^ab^	10.3	87.4	8.64
SEM		7.46	0.15	0.40	0.04	9.16	0.15	0.26	0.06
*p*-value		0.003	0.101	0.484	0.079	0.019	0.462	0.097	0.922

The means and pooled standard errors of tibia and femur breaking strength were calculated from raw untransformed data, while statistical analysis was performed using log-transformed data. ^a,b,c^ Means within columns not sharing a common suffix are significantly different at the 5% level of probability. SP: A standard protein diet contains 15.8% crude protein. RP: A reduced protein diet contains 13.8% crude protein. RP-Arg low, RP-Cit low, RP-GAA low: RP diet supplemented with Arg, Cit and GAA at the low level (0.06% or 0.6 g/kg), respectively. RP-Arg high, RP-Cit high, RP-GAA high: RP diet supplemented with Arg, Cit and GAA at the high level (0.12% or 1.2 g/kg), respectively.

## Data Availability

The original contributions presented in this study are included in the article. Further inquiries can be directed to the corresponding author.

## Supplementary Materials

The following supporting information can be downloaded at: https://www.mdpi.com/article/10.3390/ani16111664/s1. Figure S1. (a) The average, maximum and minimum temperature of the layer hen house from 60 to 75 weeks of age. (b) Relative humidity (%) of the layer hen house from 60 to 75 weeks of age.
