# Supplementary material for: The Effects of Arginine, Guanidinoacetic Acid and Citrulline Supplementation to Reduced Protein Diets for Aged Laying Hens
Source: Animals (Basel). 2026 May 29;16(11):1664. doi: 10.3390/ani16111664 (PMC13255682; doi:10.3390/ani16111664)
Supplement: Supplementary file 1 [file animals-16-01664-s001.zip › animals-4306313-supplementary.pdf]

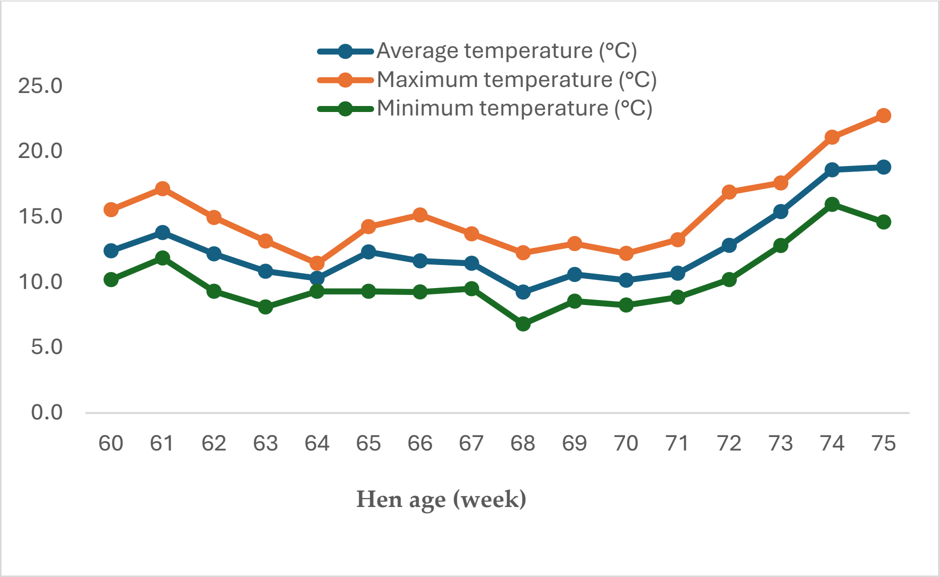

Figure S1.a. The average, maximum and minimum temperature of the layer hen house from 60 to 75 weeks of age.

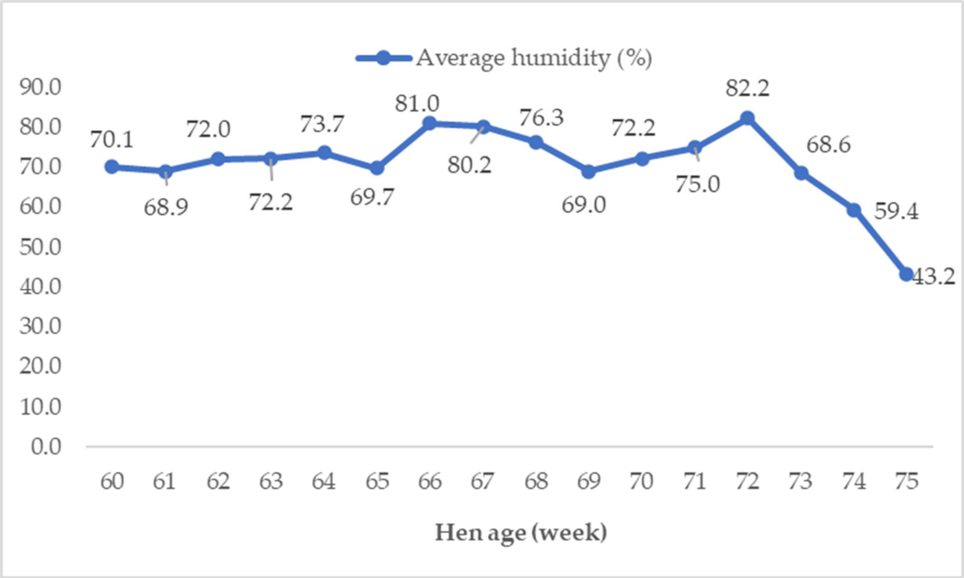

Figure S1.b. Relative humidity (%) of the layer hen house from 60 to 75 weeks of age.
